# Number of medications and adverse drug events by unintentional poisoning among older adults in consideration of inappropriate drug use: a Swedish population-based matched case-control study

**DOI:** 10.1007/s00228-017-2220-8

**Published:** 2017-03-09

**Authors:** Christian Rausch, L. Laflamme, U. Bültmann, J. Möller

**Affiliations:** 1 Department of Public Health Sciences, Karolinska Institutet, Widerströmska huset 4:th floor, Tomtebodavägen 18A, SE 17177 Stockholm, Sweden; 2 Department of Health Sciences, Community and Occupational Medicine, University of Groningen, University Medical Center Groningen, Antonius Deusinglaan 1, FA10, 9713 AV Groningen, The Netherlands

**Keywords:** Adverse drug events, Older, Elderly, Poisoning, Polypharmacy

## Abstract

**Purpose:**

This national, population-based study aims to determine the association between the number of prescribed medications and adverse drug events (ADE) by unintentional poisoning and examine this risk when known indicators of inappropriate drug use (IDU) are accounted for.

**Methods:**

We employed a matched case-control design among people living in Sweden who were 50 years and older. Cases experiencing an ADE by unintentional poisoning resulting in hospitalization or death (*n* = 5336) were extracted from the National Health and Death Registers from January 2006 to December 2009. Four controls per case matched by age, sex and residential area were randomly selected among those without an ADE (*n* = 21,344). Prescribed medications dispensed during the 4-month period prior to the ADE were identified via the Swedish Prescribed Drug Register and coded according to the number of different dispensed medications (NDDM) (0 to 10 medications) and IDU indicators (one single-drug, and three drug-combinations). Conditional logistic regression was used.

**Results:**

Each of the IDU indicators was significantly associated with very high risks of ADE. For NDDM, we found a lower but graded positive association from *two* to ten or more medications (adjusted OR, 1.5; 95% CI, 1.2–1.8). Exclusion of IDU from the NDDM decreased the risk of ADE, but the effects remained significant for *three* or more medications (adjusted OR excl. IDU, 1.5; 95% CI, 1.2–2.0).

**Conclusion:**

At population level, the number of different dispensed medications starting from three or more remains associated with ADE even after adjusting for known IDUs. Clinicians and patients need to be made aware of the increased likelihood of serious ADE, not only in case of documented inappropriate medications but also in the case of an increasing number of medications.

**Electronic supplementary material:**

The online version of this article (doi:10.1007/s00228-017-2220-8) contains supplementary material, which is available to authorized users.

## Introduction

Medications are essential for treatment of several health conditions. Among older people, as comorbidities are common, the number of prescribed medications is often high [1] and that may introduce a risk for adverse events [2, 3]. The risk may arise due to either specific medications (e.g., opioids [4]), acknowledged inappropriate combinations of them [5], or their total number [5, 6]. One well documented serious type of medication-related adverse event is that of being involved in an injury, like a fall or a road traffic crash [6–9]. Unintentional poisonings or toxic effects are another type of adverse drug event (ADE) less documented but there is a growing body of epidemiological evidence on its relation to prescribed medication use among older people [10–12]. ADE deserve greater attention due to their potentially serious short- and long-term consequences, particularly in light of the increased frailty and longer recovery time among elderly [5, 12, 13].

There are in fact several physiological aging changes contributing further to the risk of ADEs. The kidney’s decrease in glomerular filtration rate can prolong the half-life of chemicals and drugs and lead to accumulated toxicity levels or unexpected drug-drug interactions [14–16]. This decrease can start as early as the age of 30–35 years [15]. A decline of liver function and a redistribution of body fat further complicate the pharmacodynamics and pharmacokinetics of medications, which in turn increase the risk of ADE [16–20]. As many age-related, metabolic changes relevant to the study of ADEs occur prior to retirement (before ± 65 years) [21, 22], broadening the age span to include adults 50 to 64 years of age is important, particular with regard to potentially inappropriate medications like opioids, sedatives, cardiovascular drugs or the combination of such [4].

Few studies have investigated ADE among older people in light of the total number of prescribed medications, when considering known inappropriate medications and combinations. Studies of the role of inappropriate drug use (IDU) in the association between multiple prescribed medications and ADEs remain scarce. Thus, in this population-based, among people of 50 years and older living in Sweden, we aim to determine the association between number of prescribed medications and ADEs, and examine this association when taking into consideration inappropriate drug use (IDU).

## Methods

### Study design

We designed a matched case-control study nested in a national cohort of 6,981,010 individuals.

### Data sources

Individuals born before 1959 and living in Sweden from 1973 onwards were identified in the Total Population Register. Data were obtained from the national health registers linked through the unique personal identification number assigned to all Swedish residents. The Swedish National Patient Register and Swedish Cause of Death Register were used to identify cases of ADE among older adults (50 years and above) during 1 January 2006 to 31 December 2009. Information on prescribed medications was extracted from the Swedish Prescribed Drug Register which is a computerized system of pharmaceutical services that records all dispensations of prescribed drugs at all pharmacies in Sweden using the five-level anatomical therapeutic and chemical classification system (ATC) [23].

### Case definition

Cases were defined as having been hospitalized or died due to ADE, coded as unintentional poisonings or toxic effect by medication and noxious substances. Hospital discharge diagnoses were used (ICD-10 codes: T36-65 as main diagnosis and X40-44,49 as external cause of morbidity) and for mortalities (ICD-10 codes X40-44,49) the underlying cause of death was used. Only the first event of ADE was considered during the study period. A total of 5336 cases were identified: 4950 as hospitalized and 386 as fatal. Date of death or admission to hospital was used as index date.

### Selection of controls

For each case, four controls were randomly selected and matched by age (month and year), sex and residential area at the index date. Individuals who did not have any ADE during the study period were eligible as controls. The total number of controls was 21,344.

### Exposure definitions

Exposure to prescribed medications was operationalized as the total number of different dispensed medications (NDDM) and dispensed prescriptions of IDU during the 4-month period prior to index date. Dispensations on the index date were not considered. Total NDDM was determined based on the 5th digit level of the ATC and categorized into 0, 1, 2, 3, 4, 5–9, 10 or more medications, with one medication serving as reference group.

We adapted seven drug-specific indicators of IDU developed by the Swedish National Board of Health and Welfare [24]. The indicators were categorized into one single-drug category and three drug-combination categories (Table 1), including the 15 most common drug-drug interactions in the Swedish population [25]. The indicators are universally applicable to the elderly Swedish population and comparable to other national guidelines on inappropriate drug use, such as the BEERS criteria and PRISCUS list [26]. The IDU indicators are not mutually exclusive and an individual can thus be exposed to several of the indicators.

**Table 1:** IDU indicators based on the definitions used by the Swedish Board for Welfare and Health [24] and Holm et al. [25]

IDU Indicator	Definition
Single drug	Received and dispensed a prescription for at least one long acting Benzodiazepine, or Tramadol, or Propiomazin, or at least one anticholinergic medication
Duplicate therapy	Received and dispensed prescriptions for two or more medications within the same ATC group
Multiple psychoactive medications	Received and dispensed prescriptions for at least three or more psychotropic medications
Drug-drug interaction^a^	Received and dispensed prescriptions for medications with known drug-drug interaction

^a^Differs from the definition used by the Swedish Board for Welfare and Health and takes into account the 15 most common drug-drug interactions in the Swedish population according to the study by Holm et al. [25]

### Confounders

Two potential confounder were considered: marital status, since previous studies have shown a protective effect of being married on other injurious ADE [27, 28] and comorbidity. Marital status was defined based on the most recent registered status in the Total Population Register at the index date and categorized in married, unmarried, divorced, widowed or unknown, and married served as the reference group. The Charlson comorbidity index (CCI) captured individual comorbidity. It distinguishes 17 comorbidities according to ICD-10 codes, each weighted from one to six [29] (a higher score represents a more severe outcome) was combined into four categories; 0, 1–2, 3–4 and 5 or more, with 0 serving as the reference group. CCI was based on hospital discharge diagnoses during the 3 years prior to the index date.

### Statistical analyses

Descriptive statistics of sex, age, marital status and comorbidity index were calculated and their distribution compared between cases and controls using Pearson chi-square tests.

The association between NDDM, IDUs and ADE was estimated by odds ratios (ORs) with corresponding 95% confidence intervals (95% CIs) using conditional logistic regression. Further, the analyses were adjusted for confounders and also stratified by age and sex. Potential confounders were individually assessed on their association to ADE. Variables with statistically significant effects (*p* value <0.05) were included in the adjusted analysis. Marital status entailed some missing data (*n* = 66, 0.002%), which were excluded from the analysis. To explore the effect of NDDM on ADE independent of IDU analysis were performed excluding individuals exposed to the IDU indicators. IBM SPSS Statistics 22 was used to perform the statistical analyses.

### Ethics

The study was approved by the Regional Ethical Review Board in Stockholm (2010/865-31/2 and 2011/15-32).

## Results

### Sample characteristics

The majority of the cases were female (56.2%), older than 64 years (72.5%) and with none or less severe comorbidity (97.6%) (Table 2). In comparison to controls, cases were less likely to be married (34.3 vs. 46.6%) and had higher comorbidity levels (CCI >0: 39.6 vs.15.5%). The most commonly prescribed medications related to fatal ADE were Zopiclone, Oxazepam, Propiomazine, Codein and Diazepam.

**Table 2:** Characteristics of the study population, stratified by case and control status, percentage (%), *n* = 26,680

Characteristics	Category	Percentage		*p* value*
		Case *n* = 5336	Control *n* = 21,344	
Sex	Male	43.8	43.8	Matched
	Female	56.2	56.2	
Age groups	50–64 years	27.5	27.5	Matched
	≥65 years	72.5	72.5	
Marital status	Married	34.3	46.6	<0.001
	Divorced	22.0	13.8	
	Widowed	29.9	29.0	
	Unmarried	13.7	10.3	
	Unknown	0.1	0.2	
Charlson comorbidity index	0	60.4	84.4	<0.001
	1–2	37.2	15.2	
	3–4	2.3	0.3	
	≥5	0.1	0.0	

*p value of Pearson chi-square tests for comparison of distribution between cases and controls

### IDU and ADE

Among cases, 50% had been exposed to IDU. All IDU indicators showed high odds ratios, with the highest effect found among those exposed to inappropriate single drugs (see Supplementary S1).

### Number of medications and ADE

In Table 3, the ORs of ADE when exposed to NDDM are presented. Proportionally more cases than controls had at least one medication during the 4 months prior to the index date (94.9 vs. 73.9%). The odds ratios increase with an increased number of medications in a dose-response manner and the effect remains after taking marital status and comorbidity into account. The highest odds ratio was seen for 10 or more medications (adjusted OR, 14.7; 95% CI, 12.1–17.8). When excluding those exposed to IDU, the risk for ADEs still remains increased for those taking three or more medications (adjusted OR, 1.5; 95% CI, 1.2–2.0).

**Table 3:** Percentages and odds ratios (OR) with 95% confidence intervals (95% CI) for ADE by number of different dispensed medications, *n* = 26,680

Number of different dispensed medications	Percentage		OR (95% CI)		
	Cases *n* = 5336	Controls *n* = 21,344	Matched^a^	Model 1^b^	Model 1 excluding those with IDU^c^
0	5.1	26.1	0.7 (0.5–0.8)	0.6 (0.5–0.8)	0.6 (0.5–0.8)
1	2.8	9.4	Ref.	Ref.	Ref.
2	3.5	8.7	1.5 (1.2–1.8)	1.4 (1.1–1.8)	1.3 (0.9–1.6)
3	4.1	8.6	1.9 (1.5–2.4)	1.9 (1.5–2.4)	1.5 (1.2–2.0)
4	5.5	7.9	3.1 (2.5–3.8)	2.9 (2.4–3.7)	2.1 (1.7–2.7)
5–9	32.2	26.7	6.0 (5.0–7.2)	5.4 (4.5–6.5)	2.8 (2.3–3.5)
≥10	46.9	12.7	20.1 (16.6–24.3)	14.7 (12.1–17.8)	3.8 (3.0–5.7)

^a^Matched by sex, age and residential area

^b^Adjusted for matching variables, marital status, and Charlson comorbidity index

^c^Excluding those with any IDU

### Stratification by age and sex

Stratification by age (younger than 65 years of age and 65 years of age or older) and sex did not reveal any significant differences between these groups (see Supplementary S2).

## Discussion

This large, population-based study confirms the existing clinical knowledge on the risk of specific medications for ADE, showing that many ADEs occur among people with prescribed medications known to be inappropriate. Yet, this study also found that about half of the ADEs did not have any IDU and among these there was a clear association with the total number of medications. The inclusion of a wider age range, individuals 50–64 rather than just 65 years and older, in this study also showed that the association between the total number of medications and ADEs is already apparent among the younger older people (>50 years).

Our study found that the risk of ADE is clearly increased by IDU prescriptions and by increasing NDDM from two medications onwards in a graded manner, even after exclusion of IDU. Our findings on the associations between IDU and multiple medications with ADE among older patients echo previous studies [3, 5, 30], but also show that the relationship between the number of medications and ADE appear already at a lower number of medications prescribed than the generally accepted polypharmacy threshold of five or more medications [3, 31].

The mechanisms for the association between NDDM and ADEs may be complex and multifaceted. In our study, about 50% of the cases of ADE did not take any medications considered to be inappropriate. Thus, unknown drug-drug interactions might be one way of explaining this, particularly since research on drug interactions between three or more medications is scarce. Undetectable interactions of prescribed medications and over-the-counter medications, herbal treatment and food substances might also add to the increased risk. Even though most of the ICD-10 codes for ADE by unintentional poisoning are defined by exposure to a specific medication, e.g., poisoning by systemic antibiotics, we found that 5.1% of the ADE cases did not have any dispensations of prescribed medication in the previous 4 months. The ADEs among these could be due to medications that were prescribed earlier, for example, with an “on-need basis.” However, this could also be due to over-the-counter medications, which our register does not account for. Decreased cognitive functions, typical among the elderly, can also lead to faulty medication use including wrong dosages or intake of someone else’s medications [32].

### Strengths and limitations

Similar approaches to identification of ADEs by unintentional poisoning, based on specific and appropriate ICD diagnoses from the hospitalization and death register, have been employed earlier [33–35]. These studies were also challenged by the use of discharge diagnoses classified according to ICD-10. Other studies have focused on specific clinical patient groups and the underlying mechanism of their diseases and prescribed medications, i.e., chronic kidney diseases [2]. Yet, it is more challenging to disentangle the role of diseases, comorbidities and medications in large population settings when the indication for prescription, which is not available in the registers, cannot be analyzed. In order to address the influence of diseases and comorbidities, we employed the Charlson comorbidity index which is mostly applicable in hospital settings when considering older adults’ chronic or severe diseases [36]. The index takes into account serious diseases that require treatment in hospital. Adjustment for this and marital status only slightly decreased the associations, but more so among those with many medications indicating that serious morbidity has been adjusted for. However, one limitation is that we were not able to adjust for morbidity not requiring hospitalization.

The main strengths of this study lie in the linkage of an individual’s information through the use of various Swedish registers, both for assessment of ADE, dispensations of medications and the potential confounders. The registers cover the entire Swedish population and are continuously updated, and as a result the study yields results that can be generalized to the entire older adult population in Sweden. By using register information, we prevent recall bias that is often a serious bias in case-control studies with retrospective information. Although the use of register information on medications has many advantages, it does not inform about actual intake or over-the-counter medications, which might lead to some degree of misclassification [37], although non-differential among cases and controls. Our study focuses on dispensations of prescribed medications, which is a better proxy for actual intake than mere prescriptions. Furthermore, none of the active ingredients related to the IDU indicators can be purchased without a prescription in Sweden. In general, the Swedish regulations are quite restrictive for the availability of medications. In settings where the access to over-the-counter medications as well as prescribed medications might be less regulated, the magnitude of the problem might be even greater.

We focused the exposure period to the 4 months prior to the index date in order to assess medications close to and current at the time of the ADE. In Sweden, medications are normally prescribed for a treatment period of 3 months, thus the chosen 4-month period would capture potential changes regarding chronic medication use that have long prescription routines. However, changes or substitutions of medications of the same kind would not be overestimated as different medications given that they lie within the same ATC code. Other pharmacoepidemiological studies using dispensation systems have shown that too narrow time intervals can result in upwards bias [38]. On the other hand, the time interval might miss medications prescribed on an according-to-need basis, leading to an underestimation of the total number of medications, though any over- or under-estimation in this setting would apply in the same manner to cases and controls.

We cannot rule out some degree of inaccuracy in reporting based on the registers as physicians might misdiagnose ADEs due to an unspecific presentation, especially among older individuals [3, 17]. Similar studies using other injurious events can rely on ICD-10 codes with more common injury manifestations as outcome, e.g., injuries due to falls as they are simpler to define and easier to categorize when clinically presented. Although attempts have been made to define ADE using ICD-10 code algorithms with acceptable sensitivity and sensibility [35], the ICD-10 codes for ADEs are scattered and unspecific, e.g., ICD-10 T50 defines ADE by diuretics and unspecified drugs, and even a more specific T50.9 still combines ADE by medications and other substances. Furthermore, ADEs can be missed, since the diagnoses in ICD-10 potentially could involve unintentional poisoning by medication without defined by sub-codes that state the involvement of a medication, e.g. E87.1 hyponatremia, a coding not used in this research, does not have any further specification on the possible involvement of a medication, but could be considered an ADE.

## Conclusion

At population level, both NDDM and IDU are associated with an increased risk of ADE by unintentional poisoning among older. The number of different dispensed medications starting from three or more remains associated with ADE even after adjusting for known IDUs. At population level, clinicians and patients need to be made aware of the increased likelihood of serious ADE, not only in case of documented inappropriate medications but also in the case of an increasing number of medications.

## Electronic supplementary material

Below is the link to the electronic supplementary material.

Supplementary S1 (DOCX 17 kb)

Supplementary S2 (DOCX 16 kb)

## Abbreviations

CCI: Charlson comorbidity index
CDR: Swedish Cause of Death Register
IDU: Inappropriate drug use
NDDM: Number of different dispensed medications
NPR: Swedish National Patient Register
SPDR: The Swedish Prescription Drug Register
