# Supplementary material for: Number of medications and adverse drug events by unintentional poisoning among older adults in consideration of inappropriate drug use: a Swedish population-based matched case-control study
Source: Eur J Clin Pharmacol. 2017 Mar 9;73(6):743–9. doi: 10.1007/s00228-017-2220-8 (PMC5423926; doi:10.1007/s00228-017-2220-8)
Supplement: Supplementary file 1 — (DOCX 17 kb) [file 228_2017_2220_MOESM1_ESM.docx]

| **Table S1.** Odds ratios (OR) with 95% confidence intervals (95% CI) for unintentional poisoning by Inappropriate Drug Use (IDU), n=26 680. | | | | | |
| --- | --- | --- | --- | --- | --- |
| IDU indicators ^a^ | Percentage | | OR (95% CI) | | |
|  | Cases n=5 336 | Controls n=21 344 | Matched ^b^ | Model 1 ^c^ | Model 2 ^d^ |
| None | 50.4 | 99.5 | Ref. | Ref. | Ref. |
| Single Drug | 38.3 | 0.4 | 171.9 (128.8–229.7) | 158.3 (118.3–212.7) | 87.7 (65.0–117.0) |
| Duplicate-Therapy | 34.9 | 0.3 | 165.1 (124.3–227.0) | 151.2 (111.8–204.3) | 79.0 (58.3–107.1) |
| Multiple Psychoactive Medications | 20.7 | 0.2 | 107.2 (78.7–146.3) | 97.3 (70.2–133.5) | 44.6 (32.3–61.3) |
| Drug-Drug Interaction ^e^ | 7.3 | 0.0 | 193.3 (96.3–390.4) | 162.7 (80.8–329.3) | 70.6 (34.8–143.4) |
| ^a^ According to definition in Table 1.  ^b^ Adjusted through matching by sex, age and residential area.  ^c^ Adjusted for matching variables, marital status and Charlson Comorbidity Index.  ^d^ Adjusted for matching variables, marital status, Charlson Comorbidity Index and number of different medications dispensed.  ^e^ Drug-drug interaction. | | | | | |

**Number of Medications and Adverse Drug Events by Unintentional Poisoning among Older Adults in Consideration of Inappropriate Drug Use: a Swedish Population-Based Matched Case-Control Study**

**European Journal of Clinical Pharmacology**

**Authors:** C Rausch ^1,2^, L Laflamme ^1^, U Bültmann ^2^, J Möller^1^

^1^ Karolinska Institutet, Department of Public Health Sciences, Stockholm, Sweden

^2^ University Medical Center Groningen, Department of Health Sciences, Community and Occupational Medicine, Groningen, The Netherlands

Christian.rausch@ki.se
