# Supplementary material for: Number of medications and adverse drug events by unintentional poisoning among older adults in consideration of inappropriate drug use: a Swedish population-based matched case-control study
Source: Eur J Clin Pharmacol. 2017 Mar 9;73(6):743–9. doi: 10.1007/s00228-017-2220-8 (PMC5423926; doi:10.1007/s00228-017-2220-8)
Supplement: Supplementary file 2 — (DOCX 16 kb) [file 228_2017_2220_MOESM2_ESM.docx]

| **Table S2.** Percentages and odds ratios (OR) with 95% confidence intervals (95% CI) ADE by number of different dispensed medications stratified by age groups and sex, n=26 680. | | | | | | |
| --- | --- | --- | --- | --- | --- | --- |
| Number of different dispensed medications | Percentage | | Model 1OR (95% CI) ^a^ | | | |
|  | Cases n=5 336 | Controls n=21 344 | Male | Female | 50 - 64 Years | ≥65 Years |
| 0 | 5.1 | 26.1 | 0.63 (0.48–0.83) | 0.56 (0.37–0.77) | 0.82 (0.62 – 1.08) | 0.37 (0.26 – 0.53) |
| 1 | 2.8 | 9.4 | Ref. | Ref. | Ref. | Ref. |
| 2 | 3.5 | 8.7 | 1.10 (0.80–1.51) | 2.02 (1.40–2.91) | 1.61 (1.17 – 2.21) | 1.25 (0.87 – 1.78) |
| 3 | 4.1 | 8.6 | 1.74 (1.29–2.35) | 2.25 (1.56–3.25) | 2.05 (1.47 – 2.84) | 1.74 (1.25 – 2.42) |
| 4 | 5.5 | 7.9 | 2.75 (2.06–3.67) | 3.40 (2.39–4.84) | 3.17 (2.29 – 4.40) | 2.67 (1.95 – 3.66) |
| 5-9 | 32.2 | 26.7 | 4.42 (3.46–5.64) | 6.96 (5.12–9.46) | 4.79 (3.66 – 6.26) | 5.08 (3.84 – 6.71) |
| ≥10 | 46.9 | 12.7 | 12.82 (9.89–16.6) | 18.4 (13.5–25.1) | 15.8 (11.6 – 21.5) | 13.3 (10.0 – 17.6) |
| ^a^ Adjusted for matching variables, civil status, occupation and Charlson Comorbidity Index. | | | | | | |

**Number of Medications and Adverse Drug Events by Unintentional Poisoning among Older Adults in Consideration of Inappropriate Drug Use: a Swedish Population-Based Matched Case-Control Study**

**European Journal of Clinical Pharmacology**

**Authors:** C Rausch ^1,2^, L Laflamme ^1^, U Bültmann ^2^, J Möller^1^

^1^ Karolinska Institutet, Department of Public Health Sciences, Stockholm, Sweden

^2^ University Medical Center Groningen, Department of Health Sciences, Community and Occupational Medicine, Groningen, The Netherlands

Christian.rausch@ki.se
